# Dietary Regimens Modify Early Onset of Obesity in Mice Haploinsufficient for *Rai1*

**DOI:** 10.1371/journal.pone.0105077

**Published:** 2014-08-15

**Authors:** Joseph T. Alaimo, Natalie H. Hahn, Sureni V. Mullegama, Sarah H. Elsea

**Affiliations:** 1 Department of Molecular and Human Genetics, Baylor College of Medicine, Houston, Texas, United States of America; 2 Department of Human and Molecular Genetics, Virginia Commonwealth University, Richmond, Virginia, United States of America; National Cancer Institute, United States of America

## Abstract

Smith-Magenis syndrome is a complex genomic disorder in which a majority of individuals are obese by adolescence. While an interstitial deletion of chromosome 17p11.2 is the leading cause, mutation or deletion of the *RAI1* gene alone results in most features of the disorder. Previous studies have shown that heterozygous knockout of *Rai1* results in an obese phenotype in mice and that Smith-Magenis syndrome mouse models have a significantly reduced fecundity and an altered transmission pattern of the mutant *Rai1* allele, complicating large, extended studies in these models. In this study, we show that breeding C57Bl/6J *Rai1^+/−^* mice with FVB/NJ to create F1 *Rai1^+/−^* offspring in a mixed genetic background ameliorates both fecundity and *Rai1* allele transmission phenotypes. These findings suggest that the mixed background provides a more robust platform for breeding and larger phenotypic studies. We also characterized the effect of dietary intake on *Rai1^+/−^* mouse growth during adolescent and early adulthood developmental stages. Animals fed a high carbohydrate or a high fat diet gained weight at a significantly faster rate than their wild type littermates. Both high fat and high carbohydrate fed *Rai1^+/−^* mice also had an increase in body fat and altered fat distribution patterns. Interestingly, *Rai1^+/−^* mice fed different diets did not display altered fasting blood glucose levels. These results suggest that dietary regimens are extremely important for individuals with Smith- Magenis syndrome and that food high in fat and carbohydrates may exacerbate obesity outcomes.

## Introduction

Smith-Magenis syndrome (SMS, OMIM 182290) is a complex genomic disorder characterized by skeletal and craniofacial anomalies, intellectual disability, sleep abnormalities, developmental delay, and a myriad of neurological and behavioral issues, including self-injurious and attention-seeking behaviors [1]. The incidence of SMS is approximately 1∶15,000–1∶25,000 and is caused by either an interstitial deletion of chromosomal region 17p11.2 encompassing the retinoic acid-induced 1 (*RAI1*) gene or a deleterious mutation of *RAI1* [2], [3]. *RAI1* is a dosage-sensitive gene that encodes a transcriptional regulator known to bind to the nucleosome core and histones [4], and haploinsufficiency of *RAI1* results in the SMS phenotype [2], [3]. Interestingly, individuals with SMS can also have a wide range of phenotypic variability [5], [6], likely due to a variety of genetic factors such as natural genetic variation modifying the phenotype, complex epistatic interactions, and the modifying effects of the environment. Previous work using mouse models has sought to understand the role of *Rai1* in SMS by characterizing the functional effects of the gene on an array of phenotypic measures; however, the etiology and pathogenicity of altered *Rai1* expression during development and on homeostatic cellular mechanisms remains unclear. SMS mouse models display a range of behavioral abnormalities, variable penetrance of craniofacial defects, obesity, and reduced fecundity [7]–[9]. The variability of some phenotypic features has been associated with modifier alleles that either mask or exacerbate particular phenotypic outcomes [8], [10], while congenic strains have been shown to display altered Mendelian transmission of mutant *Rai1* alleles [10].

Interestingly, 90% of individuals with SMS exhibit an early onset of obesity prior to or during their adolescent years, which persists throughout their adult life [11]. Previous studies have shown that a significant number of adolescent individuals with either a deletion or point mutation of *RAI1* present predominantly with truncal-abdominal obesity or are in the 90^th^ percentile or greater for weight [11], [12]. Abdominal obesity confers an increased risk for a variety of obesity-related comorbidities such as hypertension, diabetes, and metabolic syndrome [13], [14]. In addition, there is an increased prevalence of hypercholesterolemia and hyperphagia in SMS [11], [12]. Despite the prevalence of compulsive behaviors in individuals with SMS, the prevalence of obesity is not always concurrent with compulsive eating behaviors [6], [11].

We sought to generate a SMS mouse model to address the issues of reduced transmission rates of *Rai1* and fecundity to facilitate larger phenotypic and therapeutic analyses. We performed breeding studies by generating *Rai1^+/−^* mice in a mixed genetic background of C57Bl/6J and FVB/NJ and characterized the offspring for the development of obesity through adolescence and adulthood. Finally, we characterized the effects of diet in *Rai1^+/−^* mice on the development of obesity and asked if dietary regimens could modify obesity outcomes. Our studies suggest that strain differences exist in the transmission rates of *Rai1* and fecundity and that the C57Bl/6J: FVB/NJ mixed genetic background provides a more robust and sensitized background for breeding studies. Additionally, we show that high carbohydrate and high fat diets significantly alter developmental growth rates and exacerbate obesity in SMS mice but do not alter fasting blood glucose levels.

## Materials and Methods

### Mouse breeding and husbandry

Mice heterozygous for an insertional mutation of *Rai1* (previously reported as B6.129s7-*Rai1* ^tm1Jrl^/J in Bi et al., 2005 are referred to here as *Rai1^+/−^*) were obtained from Jackson Laboratories (stocks #005981) as a fully backcrossed strain (with C57Bl/6J-Tyr^c-2J^/J). *Rai1^+/−^* mice were bred in-house with C57Bl/6J mice and FVB/NJ (Jackson Laboratories, stock #001800) mice creating F1 progeny that were C57Bl/6J:FVB/NJ mice. Pups were weaned at at 4–5 weeks due to smaller birth size and failure to thrive for *Rai1^+/−^* animals. Therefore, all pups were kept with mothers and weaned at the same age but were weaned to normal chow by 5 weeks. Animals had access to water and chow *ad libitum* [11]. The mating scheme comprised both male and female mice from all genotypes. For breeding, male mice were placed in a new cage and allowed to establish home-cage territory. After 24–48 hours, 1–2 female mice aged between 8–16 weeks were added to the cage for mating [15]. As standard practice, experienced female mice were also added to cages either during the mating process or near the end of gestation (process called “aunting”) to improve survival for all litters.

### Genotyping

Mice were genotyped as previously described [11]. Briefly, DNA was extracted from mouse tails using a standard phenol/chloroform procedure. PCR was carried out using P1F and P1R primers for identification of wild type and *Rai1^+/−^* mice as described in Bi *et al.* (2005).

### RNA Isolation

Total RNA was isolated as previously described [11], [16]. Briefly, whole liver was homogenized in TRIzol (Life Technologies) according to the manufacturer's protocol. RNA concentration and quality (260/280 ratio) was determined for each sample using a NanoDrop 1000 spectrophotometer (Thermo Scientific).

### Quantitative Real-Time PCR

Gene expression of *Rai1* in liver for each mouse was performed as previously described [11], [16]. Briefly, 2 µg of total RNA underwent first-strand cDNA synthesis using qScript (Quanta Bioscience) according to the manufacturer's protocol. Predesigned TaqMan probes (Life Technologies) for mouse mRNA expression were used to detect gene expression of *Rai1* (Mm01163529_m1) and *Gapdh* (mM99999915_g1). All samples were run in triplicate in 10 µL reaction volumes using TaqMan Universal PCR Master Mix (Life Technologies) in an ABI Prism 7900HT Sequence Detection System. Relative differences in transcript levels were quantified using the Livak Method with *Gapdh* mRNA as an endogenous control. All expression values were calculated relative to controls levels set to 1.

### Feeding Studies

Female mice were fed one of three types of food for the duration of the study. Animals were fed normal chow (NC: 21.5% protein, 23.4% fat, 55% carbohydrate; Lab Diet 5P06 Prolab RMH 2000), high-carbohydrate chow (HC: 17.8% protein, 11.8% fat, 70.4% carbohydrate; Teklad TD.98090), or high fat/low-carbohydrate chow (HF: 16.4% protein, 58% fat, 25.5% carbohydrate; Open Source D12331) and given access *ad libitum* at a constant temperature (21°C), humidity (40%) and an automatic 12 hour light/dark cycle. Initial weights were recorded for each mouse before commencing diet regimens and taken weekly thereafter. Mice were anesthetized using aerosolized isoflurane prior to weighing. All animals were housed in compliance with the guidelines of the IACUC at Virginia Commonwealth University.

### Fat Distribution

Fat pads in mice were collected and fat distribution analyzed as previously described but with minor modifications [11]. Briefly, 16–18 week old aged mice were weighed and euthanized by aerosolized isoflurane treatment, followed by a chest cavity puncture to minimize suffering. Fat was collected from both intra-abdominal (gonadal, retroperitoneal, and mesenteric) and subcutaneous (dorsal, inguinal, and groin) fat pads by dissection and weighed. Each fat was weighed separately and then combined to obtain the total body fat collected. During dissection, we observed that all mice had small, translucent cysts within the folds of the center of the liver. Cysts were observed across all genotypes.

### Blood Glucose

Blood glucose levels were assessed as previously described [11]. Briefly, animals were fasted for at least 12 hours prior to measuring blood glucose, and blood was drawn from animals by needle prick at either the end of the tail or cheek. Blood samples were measured for glucose using capillary test strips and a glucometer (EasyPRO).

### Statistical Analysis

All statistical analyses were performed using GraphPad Prism Version 6 software. Multiple comparisons were performed using an ANOVA and Bonferroni post hoc correction. Single comparisons were performed using a standard unpaired t-test. Statistical significance was determined at *P*≤0.05. All figures were generated using GraphPad Prism Version 6 software.

Data analysis for the growth studies was done by measuring the slope of the line for each individual animal during weeks 5–9 and weeks 10–16. Significance was determined by comparing the average slope between strains within each treatment group during each developmental stage using a standard unpaired t-test. Weight change for each development stage was determined by subtracting the ending weight from the beginning weight. Significance was determined by comparing the average weight change between strains within each treatment group during each developmental stage, as described.

## Results

### *Rai1^+/−^* mice have improved fecundity in a C57Bl/6J:FVB/NJ mixed genetic background

**Figure 1:**
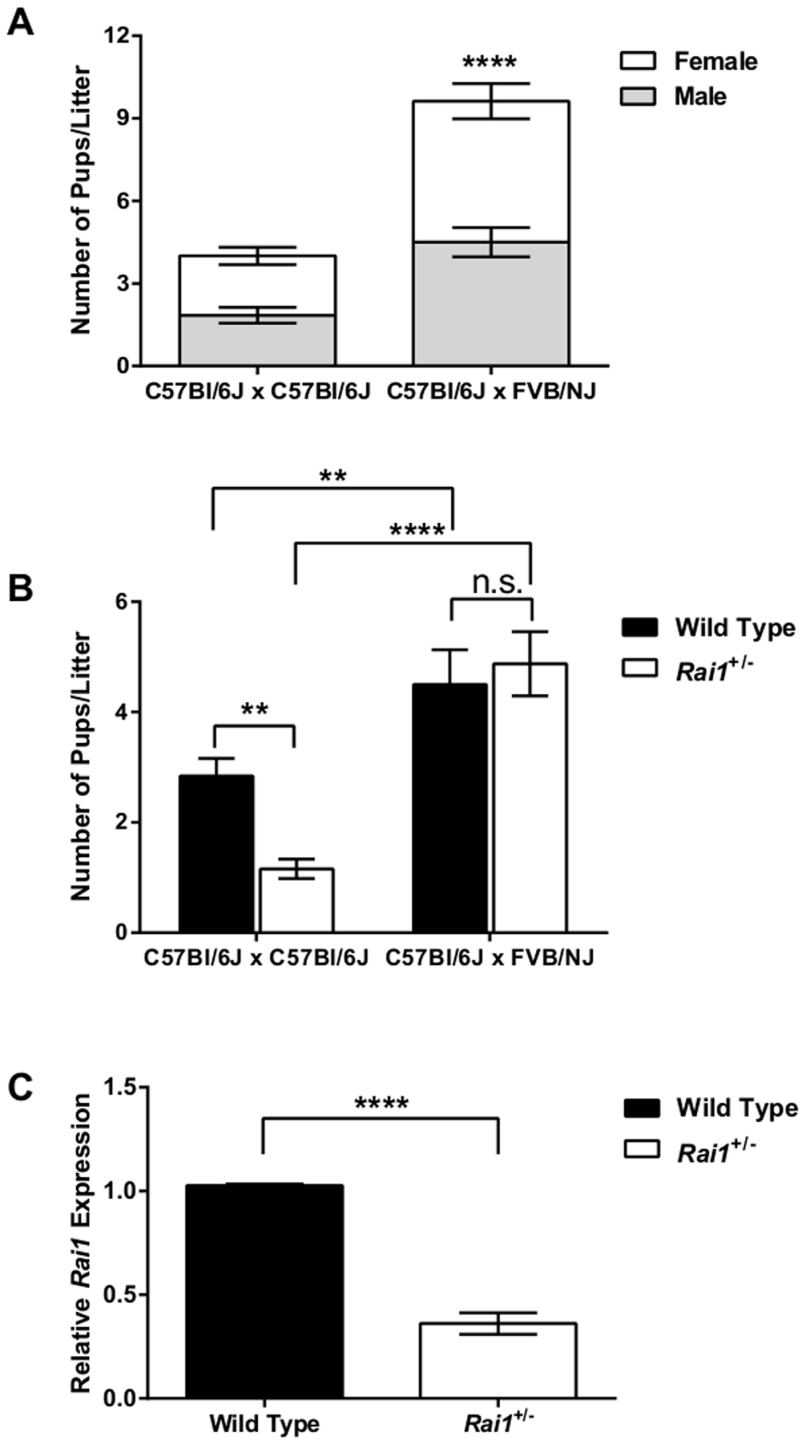
*Rai1^+/−^* mice have improved fecundity in a FVB/NJ genetic background. (**A**). *Rai1^+/−^* mice in the C57Bl/6J background when mated to C57Bl/6J produced significantly fewer progeny relative to *Rai1^+/−^* mice mated to a FVB/NJ genetic background. In either background, the proportion of male and female progeny was not significantly different. No significant difference was observed for transmission of the *Rai1^+/−^* allele by either parent (data not shown). (**B**). The number of *Rai1^+/−^* progeny produced in a C57Bl/6J genetic background is significantly less than the number of wild type progeny produced, indicating altered Mendelian ratios; however, the number of *Rai1^+/−^* progeny produced when mating occurs in the FVB/NJ genetic background is not significantly different than the number of wild type progeny, consistent with Mendelian transmission. Number of litters: C57Bl/6J x C57Bl/6J n = 19; C57Bl/6J x FVB/NJ n = 8, with either parent carrying the *Rai1^+/−^* allele. (**C**). C57Bl/6J:FVB/NJ mixed background mice that are heterozygous at the *Rai1* locus have significantly reduced *Rai1* expression. All data are plotted as mean +/− SEM; n.s. = not significant; **P<0.01; ***P<0.001; ****P<0.0001, WT n = 8, *Rai1^+/−^* n = 8.

Previous studies have shown that breeding *Rai1* ^+/−^ mice solely in a C57Bl/6J background results in a distorted Mendelian transmission [15]. We sought to ask if this skewed transmission was unique to the C57Bl/6J genetic background and thus, bred *Rai1^+/−^* mice with wild type FVB/NJ mice to create an F1 generation in the C57Bl/6J:FVB/NJ genetic background. *Rai1* ^+/−^ mice when bred in the congenic C57Bl/6J background produced an average of 4 animals per litter; however, C57Bl/6J *Rai1^+/−^* mice when mated to wild type FVB/NJ genetic background saw a significant improvement in fecundity (*P*<0.0001) with an average of 10 pups per litter (Figure 1A). Next, we genotyped all pups from both breeding experiments and found that a pure C57Bl/6J background yielded significantly fewer *Rai1* ^+/−^ mice than wild type mice (*P* = 0.0008) (Figure 1B). Additionally, C57Bl/6J mice bred into the FVB/NJ background produced significantly more wild type (*P* = 0.0075) and *Rai1* ^+/−^ (*P*<0.0001) pups compared to a pure C57Bl/6J background (Figure 1B), with an equal distribution of wild type and *Rai1* ^+/−^ pups (Figure 1B).

Next, we asked if the genetic background affected the transmission rates of the *Rai1* mutant allele. We found that mating in a pure C57Bl/6J background resulted in a significant deviation from the expected Mendelian ratio (χ^2^ = 13.47, *P*<0.001), confirming our previous results [15]. However, genotyped animals from a mixed C57Bl/6J:FVB/NJ background did not have a significant deviation from the expected Mendelian ratio (χ^2^ = 0.12, *P*>0.05) (Table 1 and Figure 1B) suggesting that a confounding genetic factor present in the C57Bl/6J background affects *Rai1* transmission rates. We quantified the level of *Rai1* expression in mixed background *Rai1* ^+/−^ mice and, as expected, found a significant reduction in mRNA levels (Figure 1C). Taken together, our breeding experiments indicate that breeding *Rai1* ^+/−^ in a pure C57Bl/6J background results in reduced fecundity, and mixing this background with FVB/NJ ameliorates fertility issues. Additionally, we were able to restore *Rai1* transmission rates to an observable Mendelian range.

**Table 1:** *Rai1* ^+/−^ breeding in the C57Bl/6J genetic background.

Male *Rai1^+/−^* x Female C57Bl/6J (average litter size = 4 pups/litter)		
Total pups	*Rai1^+/−^*	Wild Type
60	14	46
Female *Rai1^+/−^* x Male C57BL/6J (average litter size = 3.5 pups/litter)		
Total pups	*Rai1^+/−^*	Wild Type
14	4	10
Male *Rai1^+/−^* C57Bl/6J x Female FVB/NJ (average litter size = 9.8 pups/litter)		
Total pups	*Rai1* ^+/−^	Wild Type
59	26	33
Female *Rai1^+/−^* C57Bl/6J x Male FVB/NJ		
Total pups	*Rai1* ^+/−^	Wild Type
9	2	7
Male *Rai1^+/−^* FVB/NJ x Female C57Bl/6J		
Total pups	*Rai1* ^+/−^	Wild Type
9	5	4

### Dietary regimens modify growth and weight changes in *Rai1^+/−^* mice in a C57Bl/6J:FVB/NJ mixed background

**Figure 2:**
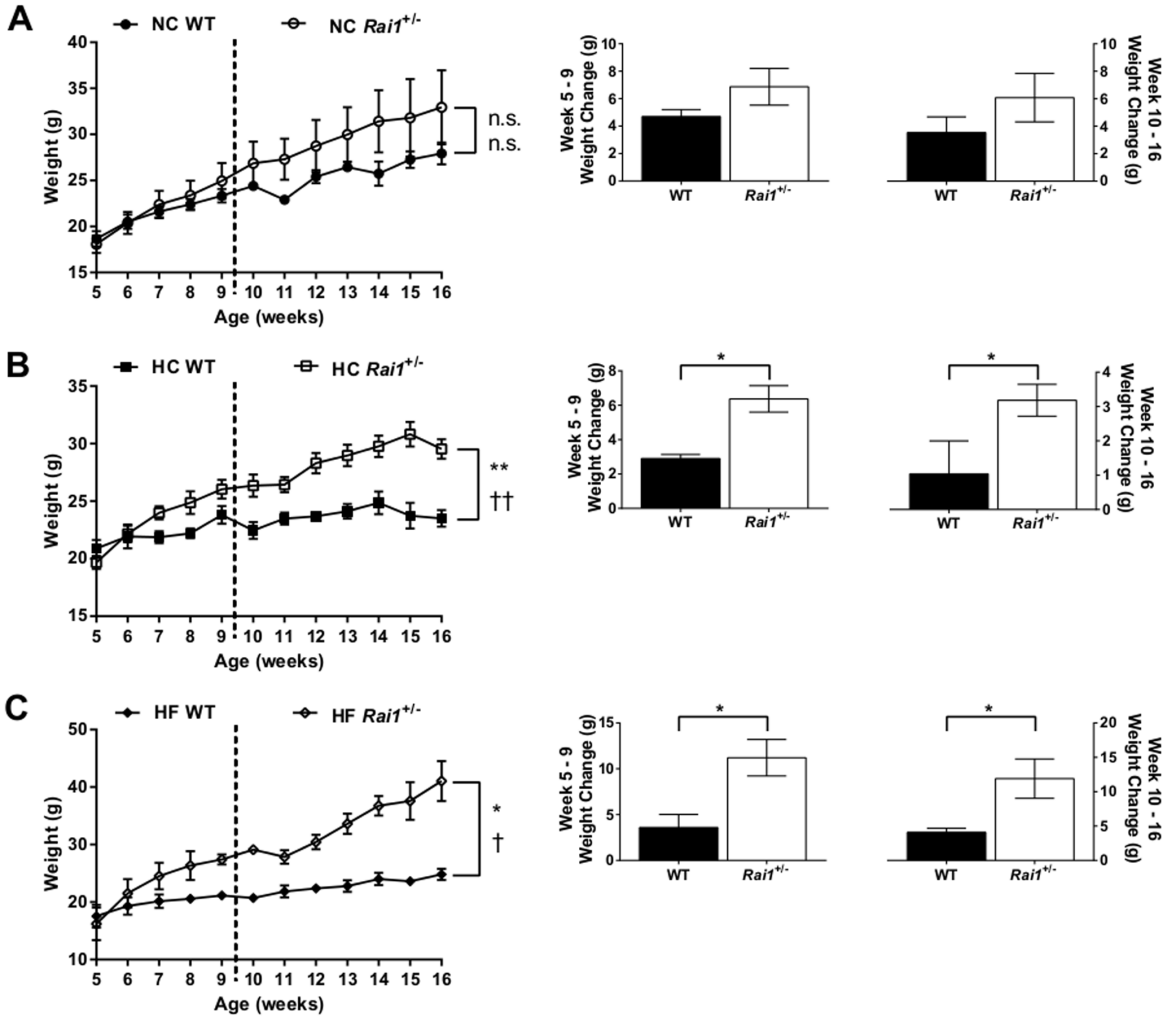
High carbohydrate and high fat diets alter growth rates in *Rai1* ^+/−^ mixed background mice. (**A**). All mice fed a normal chow diet had similar growth rates during adolescence (weeks 5–9) or adulthood (weeks 10–16). No significant difference was observed in the amount of weight gained during each developmental stage. (**B**). *Rai1^+/−^* mice fed a high carbohydrate diet had significantly faster growth during adolescence and adulthood relative to wild type mice. *Rai1^+/−^* mice gained significantly more weight during adolescence and adulthood relative to wild type. (**C**). *Rai1^+/−^* mice grew significantly faster and gained more weight when fed a high fat diet during both adolescent and adult stages of development relative to wild type mice. All data are plotted as means +/− SEM; dashed line represents the separation between developmental stages. Left panel; adolescence (weeks 5–9); n.s. = not significant; *P<0.05; **P<0.01; adulthood (weeks 10–16); n.s. = not significant; † *P*<0.05; †† *P*<0.01. Right panel; * *P*<0.05. NC: WT n = 4, *Rai1^+/−^* n = 8. HC: WT n = 3, *Rai1^+/−^* n = 7. HF: WT n = 4, *Rai1^+/−^* n = 3.

About 90% of individuals with SMS are obese by their adolescence, and obesity persists throughout their adulthood [6], [11], [12]. Previous work has also shown that SMS mouse models exhibit significant weight gain during development and are also obese [10], [11], [17]. We characterized the effects of three content-specific diets on the growth rate of *Rai1* ^+/−^ animals in the C57Bl/6J:FVB/NJ mixed background during two stages of development; adolescence and early adulthood. Mice were fed either normal, high carbohydrate, or high fat specific diets starting at week 5, and growth was measured during their adolescent stage (weeks 5–9) and during early adulthood (weeks 10–16). *Rai1* ^+/−^ animals fed normal chow did not significantly differ for rate of growth during either developmental stage (adolescence *P* = 0.2645; early adulthood *P* = 0.5058) (Figure 2A) and did not have differences in weight gained during both periods (adolescence *P* = 0.2962; early adulthood *P* = 0.3635) relative to wild type normal chow fed mice (Figure 2A). Previous studies have shown that *Rai1* ^+/−^ mice gain significantly more weight than wild type mice by week 20 on standard laboratory chow, and this weight gain continued into adulthood [7], [10], [11]. However, we did not look beyond the 16 week treatment period in this study, and therefore, it is possible to speculate that these animals may have displayed significant weight gains at later time points. Interestingly, *Rai1* ^+/−^ animals fed a high carbohydrate diet gained significantly more weight (Figure 2B) and gained weight at a faster rate than wild type animals during both adolescence (weight gained *P* = 0.0221; rate of growth *P* = 0.0024) and throughout early adulthood (weight gained *P* = 0.05; rate of growth *P* = 0.0076) relative to high carbohydrate fed wild type mice (Figure 2B). We also observed similar results for *Rai1* ^+/−^ animals fed a high fat diet. *Rai1^+/−^* haploinsufficient mice had significantly more growth during both developmental stages relative to high fat fed wild type mice (adolescence *P* = 0.02; early adulthood *P* = 0.0193) (Figure 2C). In addition, *Rai1^+/−^* mice gained more weight during those time periods (adolescence *P* = 0.0236; early adulthood *P* = 0.0257) (Figure 2C).

### Dietary regimens modify abdominal and subcutaneous fat distribution patterns but not fasting blood glucose levels in *Rai1^+/−^* mice in a C57Bl/6J:FVB/NJ mixed background

**Figure 3:**
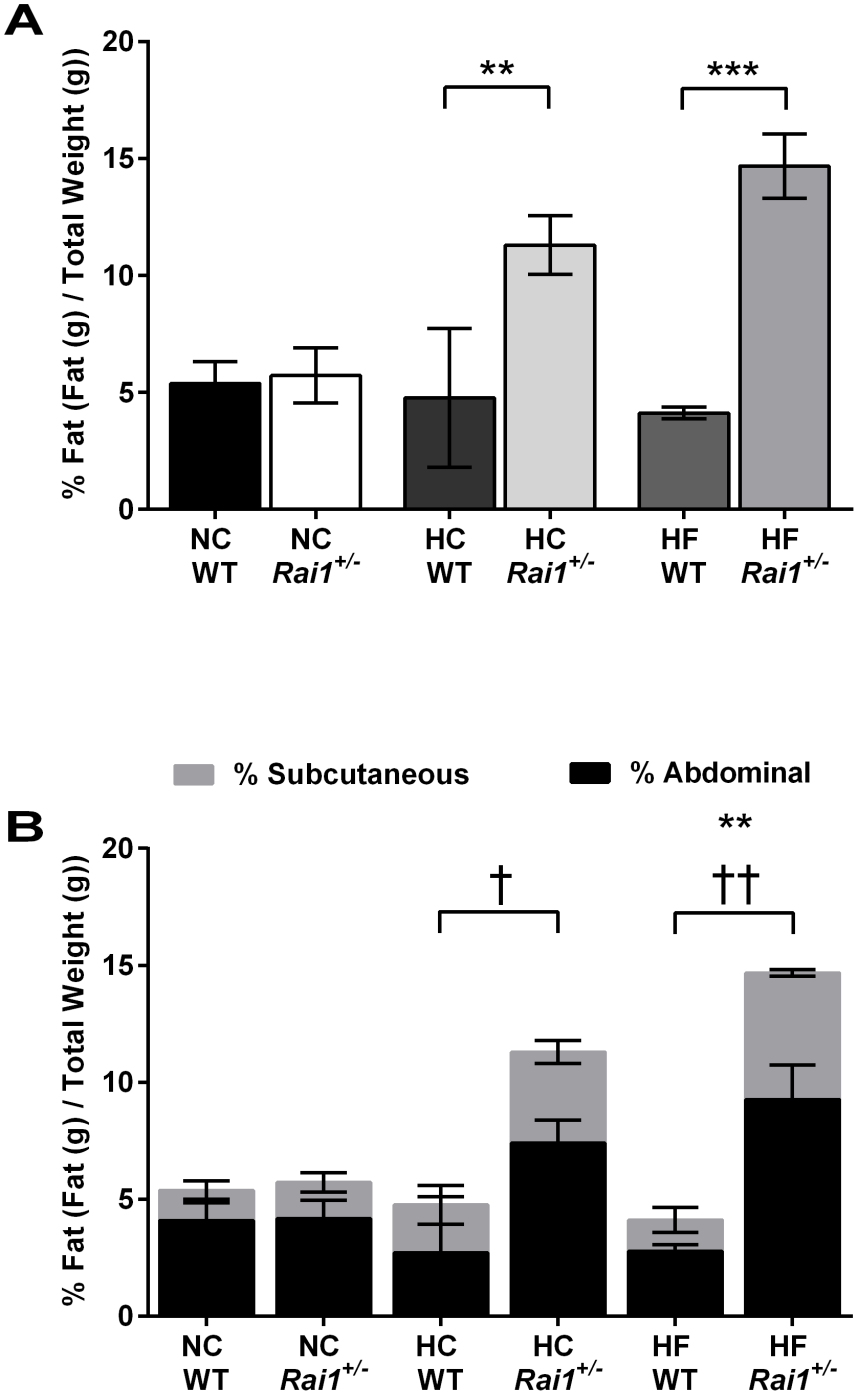
High carbohydrate and high fat fed *Rai1* ^+/−^ mice have altered body fat and fat distribution. (**A**). Wild type and *Rai1^+/−^* mice fed normal chow did not have significantly different total body fat. Both high carbohydrate and high fat fed *Rai1^+/−^* mice had significantly more body fat than high carbohydrate and high fat fed wild type mice. (**B**). *Rai1* ^+/−^ mice on a high fat diet had significantly more subcutaneous and abdominal fat relative to high fat fed wild type mice. However high carbohydrate fed *Rai1* ^+/−^ mice only displayed alterations to abdominal fat portions but not subcutaneous. Normal chow diet regimen did not alter the distribution of fat in either genotype. All data are plotted as means +/− SEM; ** *P*<0.01; *** *P*<0.001; † *P*<0.05; †† *P*<0.01. NC: WT n = 3, *Rai1^+/−^* n = 3. HC: WT n = 2, *Rai1^+/−^* n = 4. HF WT n = 3, *Rai1^+/−^* n = 3.

**Figure 4:**
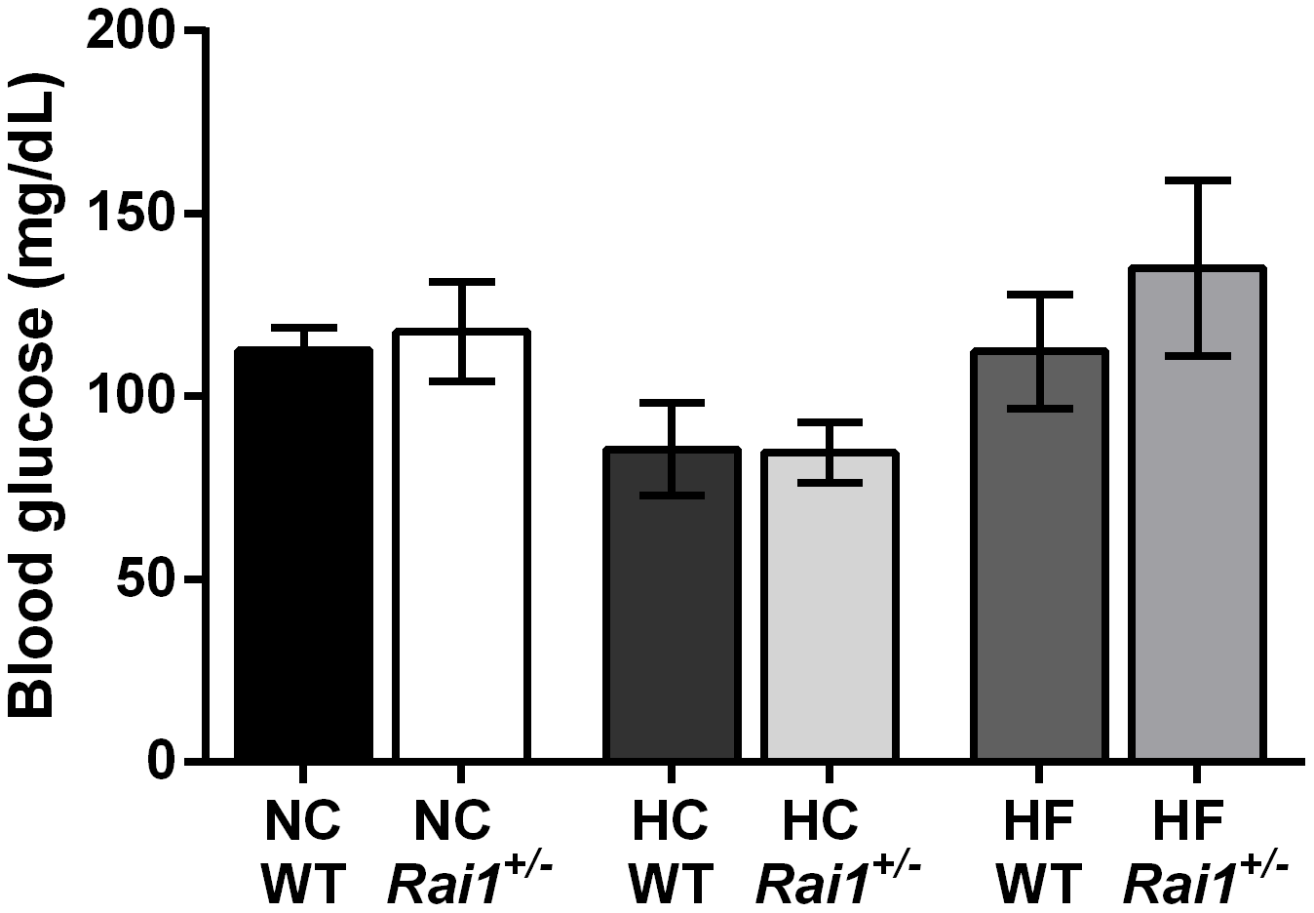
Blood glucose levels are not altered due to diet regimens. Mice fed either normal chow, high carbohydrate, or high fat do not have altered blood glucose levels after 12 hours of fasting. All data are plotted as mean +/− SEM. NC: WT n = 5, *Rai1^+/^* ^−^n = 8. HC: WT n = 3, *Rai1^+/−^* n = 7. HF: WT n = 3, *Rai1^+/−^* n = 5.

Next, we characterized each mouse for adiposity and fat distribution. *Rai1* ^+/−^ animals fed normal chow did not have any significant differences in percentage of body fat (*P*>0.9999) (Figure 3A) or distribution of fat (abdominal *P*>0.9999; subcutaneous *P*>0.9999) (Figure 3B) relative to normal chow fed wild type mice. *Rai1* ^+/−^ animals fed a high carbohydrate diet displayed a significant increase in the percentage of body fat (*P* = 0.0181) relative to high carbohydrate fed wild type mice. Interestingly the high carbohydrate fed *Rai1* ^+/−^ animals also displayed a significant increase in the percentage of abdominal fat (*P* = 0.0387), but not a significant increase in the percentage of subcutaneous fat (*P* = 0.0725). *Rai1^+/−^* animals fed a high fat diet had a significantly higher percentage of body fat (*P* = 0.0003), and these animals had significantly more fat in each the subcutaneous (*P* = 0.0002) and abdominal (*P* = 0.0032) fat pads relative to wild type high fat fed animals. An increase in fat tissue is known to be associated with insulin resistance, cardiovascular disease, cancers, and hypertension [14]. Therefore, we sought to characterize any alterations to basal metabolic function in each diet fed mice by measure fasting blood glucose levels. Fasting blood glucose levels were not altered by diet in either genotype (Figure 4).

## Discussion

Phenotypic variability is observed across a variety of genomic disorders including SMS, Potocki-Lupski syndrome (OMIM 610883), Angelman syndrome (OMIM 105830), and brachydactyly-mental retardation syndrome (OMIM 600430) [5], [18]. Individual differences in the genome that act to modify phenotypic outcomes of these particular disorders are likely one of many factors contributing to the range of clinical findings. The mechanisms in which polymorphisms or copy number variation in the genome exert their effects is unclear but are thought to either exacerbate or reduce the severity of phenotypic features, thus resulting in a spectrum of phenotypic features across disorders and increasing the difficulty of a clinical diagnosis.

In evaluating genomic disorders, mouse models have become paramount in dissecting the role of the causative genes in cellular, molecular, and behavioral processes. However, not all phenotypes in humans are recapitulated in mice and therefore some studies are more focused on particular subsets of phenotypes or pathways of the disorder. Generating and characterizing a mouse model is time consuming, and one major concern is the effect of the genetic background on the interpretation of the studies [19], [20]. Differences in genetic background have been shown to contribute greatly to the degree of phenotypic variability in mice studies. For example, *Fmr1* knockout mice bred on a mixed FVB/129 background had significant learning deficits, whereas *Fmr1* knockout mice bred on a B6/129 background did not [21]. In addition, mouse models of DYT1 dystonia have shown that genetic background modulates lifespan and motor function indicating that modifying alleles can contribute to the heterogeneity found in a phenotype [22].

The results from our breeding experiments presented here indicate that haploinsufficiency of *Rai1* in a pure C57Bl/6J background results in a severe alteration in Mendelian transmission rates. These data would suggest that the congenic strain may carry recessive alleles that together create an adverse genetic event resulting in a reduced transmission of *Rai1* (Figure 1A–B and Table 1). Other studies have shown that the more congenic the C57Bl/6J background becomes, the inheritances rates of *Rai1* are significantly reduced [15]. Furthermore, mouse fecundity is also severely affected, and previous literature has reported breeding difficulties with *Rai1^+/−^* mice [7]–[9]. When mating *Rai1^+/−^* in a pure C57Bl/6J background, as few as 4 pups per litter were born, consistent with previous findings (Figure 1A) [15]. Crossbreeding the C57Bl/6J mice with FVB/NJ resulted in improved fecundity of *Rai1^+/−^* mice, producing on average 10 pups per litter (Figure 1A–B) and restored transmission ratios within an expected Mendelian range. These results lend further evidence that the expression of one or more recessive modifier genes in the C57Bl/6J background influences *Rai1* transmission and animal fecundity.

Truncal-abdominal obesity is observed in a majority of SMS patients during early adolescence and other SMS mouse models have shown obesity phenotypes, further implicating *RAI1* as a contributor to obesity and body weight [7]–[9], [11], [23], [24]. Furthermore, there is a well-established body of evidence implicating chromatin-modifying proteins in the development of obesity. In our analysis, we focused on growth and the development of obesity during adolescence and early adulthood under different content-specific diets. *Rai1^+/−^* mice fed normal chow did not have significant weight gain during either period of feeding (Figure 2A). However, other studies have reported an obese phenotype in *Rai1^+/−^* mice when fed normal chow as early as week 20. We predict that the lack of an obesity phenotype observed in our data in general is not solely due to genetic background, but likely due to the restricted timeline used for observing the phenotype. Furthermore, other SMS mouse studies have not reported any significant weight changes in mice fed normal chow prior to week 20 except very early in development at week 5, but not during weeks 6–19 [17]. When *Rai1^+/−^* mice are fed a high carbohydrate diet or high fat diet, the rate of growth and the amount of weight gained during both developmental periods is significantly altered (Figure 2B–C), suggesting that these diets can exacerbate growth and weight gain when *Rai1* dosage is decreased. We then explored the increase in weight in all treated animals and found that both high carbohydrate-fed and high fat-fed mice had significant increases in the percentages of body fat, suggesting that the increase in weight was likely stored as excess fat (Figure 3A). When analyzing adiposity patterns, we found that high fat-fed *Rai1* ^+/−^ mice had significant increases in both abdominal and subcutaneous fat where as high carbohydrate-fed *Rai1* ^+/−^ animals only displayed a significant increase in abdominal fat, but a trend towards significance for subcutaneous fat.

In order to determine if these mixed background *Rai1^+/−^* mice expressed any obesity associated comorbidities, and it is well established that truncal adipose tissue has a significant impact on the development of insulin resistance [13], we assessed fasting glucose levels. We did not observe any altered fasting blood glucose levels in these mice despite treatment with different diets (Figure 4), suggesting that obesity associated insulin resistance is not present. These findings are consistent with previously reported studies that show *Rai1^+/−^* mice fed normal chow when obese at week 30 do not display insulin resistance and that despite the increase in abdominal adiposity, there was an absence of phenotypic measures resembling metabolic syndrome [11]. It is interesting that *Rai1^+/−^* mice have increased abdominal adiposity but do not manifest insulin resistance. Studies have suggested that individuals who are obese but metabolically healthy are still at a significant risk for cardiovascular events and mortality relative to non-obese individuals [25], [26]. However, a recent study has shown that mice carrying a chromosomal deletion of *Rai1* (and several other genes) commonly found amongst individuals with SMS display a range of phenotypic features manifesting metabolic syndrome. These mice were found to have an increase in weight and fat and a decrease in high-density lipoprotein levels and insulin sensitivity [17]. These studies have suggested that another gene within the deletion region, *Srebf1*, may act additively with *Rai1* causing the observed endophenotype [17]. Additionally, the susceptibility of both C57Bl/6J to diet induced obesity or insulin resistance has been reported [27], [28]. However, while data show that FVB/NJ mice are not susceptible to diet induced obesity, studies have shown that particular mutations in the FVB/NJ result in an obesity phenotype in addition to increased glucose levels when fed a normal diet [27]–[29].

A growing body of evidence suggests that obesity is about 65% genetic and that gene-environment interactions may confer an even greater susceptibility to obesity [30]. One leading hypotheses is that early environmental influences may induce epigenetic variation at specific loci that result in permanent defects in metabolism and increases in disease risk [31]. Three genes, *MC4R, LEP* and *POMC*, have been identified through genetic association to confer an increase in obesity susceptibility and are also environmentally responsive to dietary intake. Previous work has shown that adult mice fed high fat diets have a decrease in methylation in whole brain tissue at the transcriptional start site of *Mc4r* [32]. Obese women who successfully lost weight after eating an 8-week low calorie diet showed lower methylation levels of *LEP* from adipose biopsies than those who did not lose weight [33]. Another study showed that high fat fed rats had an increase in methylation at the *Lep* promoter region in adipose tissue [34]. Rats that were overfed during neonatal development had a significant increase in methylation of the promoter region of *Pomc* in the hypothalamus [35]. Taken together, these data support environmental factors such as dietary content or food intake may have an effect on the epigenetic regulation of genes involved in obesity. While our study did not determine if the observed weight gains of mice fed either a high fat or a high carbohydrate diet exhibited any epigenetic changes, a recent study showed that *RAI1* binds to the nucleosome core and histones [4] and may impact epigenetic regulation of gene transcription. No epigenetic studies have been performed on *Rai1* haploinsufficient mice; however, our previous work has shown that *Rai1* haploinsufficient mice have a basal decrease in *Bdnf* and *Pomc* expression and an increase in *Mc4r* expression, lending evidence to the global effects *Rai1* alone has on obesity and weight pathways [11]. Future studies delineating the epigenetic profile of *Rai1* ^+/−^ mice relative to wild type mice would reveal the susceptibility of specific genomic loci to epigenetic changes that are *Rai1* dependent. Additionally, epigenetic profiles after treatment of high carbohydrate and high fat diets may uncover other loci that are environmentally responsive when *Rai1* dosage is decreased.

In summary, we present evidence that breeding *Rai1^+/−^* mice in a mixed genetic background results in improved fecundity and expected Mendelian transmission rates compared to the C57Bl/6J congenic strain, providing an additional model in which to study SMS and the role for *Rai1* in the development of obesity. In addition, we show that a high carbohydrate or high fat diet from weaning can significantly impact the rate of growth, weight gain, adiposity, and fat distribution in these animals, suggesting that dietary regimen in persons with SMS should be monitored for fat and carbohydrate content while striving to maintain a balanced diet during early development. It is possible that individuals with SMS may benefit from nutritional counseling to help reduce the risk of significant weight gain and potentially the development of obesity.

## References

[1] Elsea SH, Girirajan S (2008) Smith-Magenis syndrome. Eur J Hum Genet 16: 412–421. 18231123 10.1038/sj.ejhg.5202009

[2] Slager RE, Newton TL, Vlangos CN, Finucane B, Elsea SH (2003) Mutations in RAI1 associated with Smith-Magenis syndrome. Nat Genet 33: 466–468. 12652298 10.1038/ng1126

[3] Vlangos CN, Yim DK, Elsea SH (2003) Refinement of the Smith-Magenis syndrome critical region to approximately 950kb and assessment of 17p11.2 deletions. Are all deletions created equally? Mol Genet Metab 79: 134–141. 12809645 10.1016/s1096-7192(03)00048-9

[4] Darvekar S, Rekdal C, Johansen T, Sjottem E (2013) A phylogenetic study of SPBP and RAI1: evolutionary conservation of chromatin binding modules. PLoS One 8: e78907. 24205348 10.1371/journal.pone.0078907 PMC3799622

[5] Girirajan S, Vlangos CN, Szomju BB, Edelman E, Trevors CD, et al (2006) Genotype-phenotype correlation in Smith-Magenis syndrome: evidence that multiple genes in 17p11.2 contribute to the clinical spectrum. Genet Med 8: 417–427. 16845274 10.1097/01.gim.0000228215.32110.89

[6] Edelman EA, Girirajan S, Finucane B, Patel PI, Lupski JR, et al (2007) Gender, genotype, and phenotype differences in Smith-Magenis syndrome: a meta-analysis of 105 cases. Clin Genet 71: 540–550. 17539903 10.1111/j.1399-0004.2007.00815.x

[7] Bi W, Yan J, Shi X, Yuva-Paylor LA, Antalffy BA, et al (2007) Rai1 deficiency in mice causes learning impairment and motor dysfunction, whereas Rai1 heterozygous mice display minimal behavioral phenotypes. Hum Mol Genet 16: 1802–1813. 17517686 10.1093/hmg/ddm128

[8] Yan J, Bi W, Lupski JR (2007) Penetrance of craniofacial anomalies in mouse models of Smith-Magenis syndrome is modified by genomic sequence surrounding Rai1: not all null alleles are alike. Am J Hum Genet 80: 518–525. 17273973 10.1086/512043 PMC1821110

[9] Walz K, Spencer C, Kaasik K, Lee CC, Lupski JR, et al (2004) Behavioral characterization of mouse models for Smith-Magenis syndrome and dup(17)(p11.2p11.2). Hum Mol Genet 13: 367–378. 14709593 10.1093/hmg/ddh044

[10] Bi W, Ohyama T, Nakamura H, Yan J, Visvanathan J, et al (2005) Inactivation of Rai1 in mice recapitulates phenotypes observed in chromosome engineered mouse models for Smith-Magenis syndrome. Hum Mol Genet 14: 983–995. 15746153 10.1093/hmg/ddi085

[11] Burns B, Schmidt K, Williams SR, Kim S, Girirajan S, et al (2010) Rai1 haploinsufficiency causes reduced Bdnf expression resulting in hyperphagia, obesity and altered fat distribution in mice and humans with no evidence of metabolic syndrome. Hum Mol Genet 19: 4026–4042. 20663924 10.1093/hmg/ddq317 PMC7714048

[12] Smith AC, Gropman AL, Bailey-Wilson JE, Goker-Alpan O, Elsea SH, et al (2002) Hypercholesterolemia in children with Smith-Magenis syndrome: del (17) (p11.2p11.2). Genet Med 4: 118–125. 12180145 10.1097/00125817-200205000-00004

[13] Patel P, Abate N (2013) Role of subcutaneous adipose tissue in the pathogenesis of insulin resistance. J Obes 2013: 489187. 23691287 10.1155/2013/489187 PMC3649613

[14] Poirier P, Giles TD, Bray GA, Hong Y, Stern JS, et al (2006) Obesity and cardiovascular disease: pathophysiology, evaluation, and effect of weight loss: an update of the 1997 American Heart Association Scientific Statement on Obesity and Heart Disease from the Obesity Committee of the Council on Nutrition, Physical Activity, and Metabolism. Circulation 113: 898–918. 16380542 10.1161/CIRCULATIONAHA.106.171016

[15] Girirajan S, Elsea SH (2009) Distorted Mendelian transmission as a function of genetic background in Rai1-haploinsufficient mice. Eur J Med Genet 52: 224–228. 19116176 10.1016/j.ejmg.2008.12.002

[16] Williams SR, Zies D, Mullegama SV, Grotewiel MS, Elsea SH (2012) Smith-Magenis syndrome results in disruption of CLOCK gene transcription and reveals an integral role for RAI1 in the maintenance of circadian rhythmicity. Am J Hum Genet 90: 941–949. 22578325 10.1016/j.ajhg.2012.04.013 PMC3370274

[17] Lacaria M, Saha P, Potocki L, Bi W, Yan J, et al (2012) A duplication CNV that conveys traits reciprocal to metabolic syndrome and protects against diet-induced obesity in mice and men. PLoS Genet 8: e1002713. 22654670 10.1371/journal.pgen.1002713 PMC3359973

[18] Potocki L, Bi W, Treadwell-Deering D, Carvalho CM, Eifert A, et al (2007) Characterization of Potocki-Lupski syndrome (dup(17)(p11.2p11.2)) and delineation of a dosage-sensitive critical interval that can convey an autism phenotype. Am J Hum Genet 80: 633–649. 17357070 10.1086/512864 PMC1852712

[19] Bucan M, Abel T (2002) The mouse: genetics meets behaviour. Nat Rev Genet 3: 114–123. 11836505 10.1038/nrg728

[20] Wolfer DP, Crusio WE, Lipp HP (2002) Knockout mice: simple solutions to the problems of genetic background and flanking genes. Trends Neurosci 25: 336–340. 12079755 10.1016/s0166-2236(02)02192-6

[21] Dobkin C, Rabe A, Dumas R, El Idrissi A, Haubenstock H, et al (2000) Fmr1 knockout mouse has a distinctive strain-specific learning impairment. Neuroscience 100: 423–429. 11008180 10.1016/s0306-4522(00)00292-x

[22] Tanabe LM, Martin C, Dauer WT (2012) Genetic background modulates the phenotype of a mouse model of DYT1 dystonia. PLoS One 7: e32245. 22393392 10.1371/journal.pone.0032245 PMC3290549

[23] Walz K, Paylor R, Yan J, Bi W, Lupski JR (2006) Rai1 duplication causes physical and behavioral phenotypes in a mouse model of dup(17)(p11.2p11.2). J Clin Invest 116: 3035–3041. 17024248 10.1172/JCI28953 PMC1590269

[24] Girirajan S, Patel N, Slager RE, Tokarz ME, Bucan M, et al (2008) How much is too much? Phenotypic consequences of Rai1 overexpression in mice. Eur J Hum Genet 16: 941–954. 18285828 10.1038/ejhg.2008.21

[25] Arnlov J, Ingelsson E, Sundstrom J, Lind L (2010) Impact of body mass index and the metabolic syndrome on the risk of cardiovascular disease and death in middle-aged men. Circulation 121: 230–236. 20038741 10.1161/CIRCULATIONAHA.109.887521

[26] Kuk JL, Ardern CI (2009) Are metabolically normal but obese individuals at lower risk for all-cause mortality? Diabetes Care 32: 2297–2299. 19729521 10.2337/dc09-0574 PMC2782994

[27] Haluzik M, Colombo C, Gavrilova O, Chua S, Wolf N, et al (2004) Genetic background (C57BL/6J versus FVB/N) strongly influences the severity of diabetes and insulin resistance in ob/ob mice. Endocrinology 145: 3258–3264. 15059949 10.1210/en.2004-0219

[28] Kim DH, Gutierrez-Aguilar R, Kim HJ, Woods SC, Seeley RJ (2013) Increased adipose tissue hypoxia and capacity for angiogenesis and inflammation in young diet-sensitive C57 mice compared with diet-resistant FVB mice. Int J Obes (Lond) 37: 853–860. 22964790 10.1038/ijo.2012.141 PMC3525796

[29] Chen N, Liu L, Zhang Y, Ginsberg HN, Yu YH (2005) Whole-body insulin resistance in the absence of obesity in FVB mice with overexpression of Dgat1 in adipose tissue. Diabetes 54: 3379–3386. 16306352 10.2337/diabetes.54.12.3379

[30] Nan C, Guo B, Warner C, Fowler T, Barrett T, et al (2012) Heritability of body mass index in pre-adolescence, young adulthood and late adulthood. Eur J Epidemiol 27: 247–253. 22426805 10.1007/s10654-012-9678-6

[31] Herrera BM, Keildson S, Lindgren CM (2011) Genetics and epigenetics of obesity. Maturitas 69: 41–49. 21466928 10.1016/j.maturitas.2011.02.018 PMC3213306

[32] Widiker S, Karst S, Wagener A, Brockmann GA (2010) High-fat diet leads to a decreased methylation of the Mc4r gene in the obese BFMI and the lean B6 mouse lines. J Appl Genet 51: 193–197. 20453306 10.1007/BF03195727

[33] Staiger H, Tschritter O, Machann J, Thamer C, Fritsche A, et al (2003) Relationship of serum adiponectin and leptin concentrations with body fat distribution in humans. Obes Res 11: 368–372. 12634431 10.1038/oby.2003.48

[34] Milagro FI, Campion J, Garcia-Diaz DF, Goyenechea E, Paternain L, et al (2009) High fat diet-induced obesity modifies the methylation pattern of leptin promoter in rats. J Physiol Biochem 65: 1–9. 19588726 10.1007/BF03165964

[35] Plagemann A, Harder T, Brunn M, Harder A, Roepke K, et al (2009) Hypothalamic proopiomelanocortin promoter methylation becomes altered by early overfeeding: an epigenetic model of obesity and the metabolic syndrome. J Physiol 587: 4963–4976. 19723777 10.1113/jphysiol.2009.176156 PMC2770159

