# Correction: Dietary Regimens Modify Early Onset of Obesity in Mice Haploinsufficient for *Rai1*

**DOI:** 10.1371/journal.pone.0114092

**Published:** 2014-11-14

**Authors:** The PLOS ONE Staff

The second author’s name is spelled incorrectly. The correct author’s name is: Natalie C. Hahn.

The correct citation is: Alaimo JT, Hahn NC, Mullegama SV, Elsea SH (2014) Dietary Regimens Modify Early Onset of Obesity in Mice Haploinsufficient for *Rai1*. PLoS ONE 9(8): e105077. doi:10.1371/journal.pone.0105077
